# How much would each researcher receive if competitive government research funding were distributed equally among researchers?

**DOI:** 10.1371/journal.pone.0183967

**Published:** 2017-09-08

**Authors:** Krist Vaesen, Joel Katzav

**Affiliations:** 1 School of Innovation Sciences, Eindhoven University of Technology, Eeuwsel, Eindhoven, The Netherlands; 2 Faculty of Archaeology, Human Origins Group, University of Leiden, Leiden, the Netherlands; 3 School of Historical and Philosophical Inquiry, The University of Queensland, St Lucia, Queensland, Australia; Northwestern University, UNITED STATES

## Abstract

Scientists are increasingly dissatisfied with funding systems that rely on peer assessment and, accordingly, have suggested several proposals for reform. One of these proposals is to distribute available funds equally among all qualified researchers, with no interference from peer review. Despite its numerous benefits, such egalitarian sharing faces the objection, among others, that it would lead to an unacceptable dilution of resources. The aim of the present paper is to assess this particular objection. We estimate (for the Netherlands, the U.S. and the U.K.) how much researchers would receive were they to get an equal share of the government budgets that are currently allocated through competitive peer assessment. For the Netherlands, we furthermore estimate what researchers would receive were we to differentiate between researchers working in low-cost, intermediate-cost and high-cost disciplines. Given these estimates, we then determine what researchers could afford in terms of PhD students, Postdocs, travel and equipment. According to our results, researchers could, on average, maintain current PhD student and Postdoc employment levels, and still have at their disposal a moderate (the U.K.) to considerable (the Netherlands, U.S.) budget for travel and equipment. This suggests that the worry that egalitarian sharing leads to unacceptable dilution of resources is unjustified. Indeed, our results strongly suggest that there is room for far more egalitarian distribution of funds than happens in the highly competitive funding schemes so prevalent today.

## Introduction

In response to growing dissatisfaction with funding allocation based on scientific peer review, there has been a surge of proposals for reform. Such reform would need to address the common complaint that grant peer review bears excessive costs [1–2], appears to be unreliable [1,3] and is subject to all sorts of biases, including, among others, biases relating to gender, affiliation, age and ethnicity [1,4–6]. Proposals include more careful selection of reviewers [7], increasing the transparency of peer review [8], statistical adjustments to panel ratings [5], combining a stripped-down peer review process with a lottery [9], and various ways of allocating grants based on an assessment of researchers rather than of grant applications [10–12]. Also, some authors have suggested eliminating peer assessment and distributing available funds among all qualified researchers [13–14].

Although the equal distribution of research money among qualified researchers would depart most from how funds are currently allocated, it could have numerous benefits: it could reduce the drop out of talented scientists, the effects of gender and other biases in grant peer review, the reinforcement of established views, the incentives to commit scientific fraud, the number of experienced researchers excluded from teaching, the oversupply of junior scientists, workplace stress and negative effects on personal and family life [13,15]. Furthermore, a baseline grant system is much cheaper than schemes that involve peer assessment and, in light of our uncertainty about the future course of science, effectively spreads our risks. Finally, such a system accommodates the worry that awarding large grants only to a happy few might be ineffective; scientific impact per dollar appears lower for large grant-holders [16] and awardees do not perform extraordinarily well when compared with rejected applicants (see the review in [17]).

Despite these benefits, policy makers have been reluctant to experiment with, let alone implement, baseline grant systems. This reluctance arguably at least in part relates to the worry that, with egalitarian sharing, research money would quickly evaporate without substantive returns, especially in costly disciplines [14]. Here we assess the extent to which this fear is justified (while mostly putting aside other worries one might have about egalitarian sharing). More specifically, we present estimates, for the Netherlands, the U.S. and the U.K., of how much each qualified researcher would receive were available competitive government research funds distributed equally among such researchers. In the case of the Netherlands—but, unfortunately, not in the cases of the U.S. and the U.K.–available data also allows us to provide estimates of the rates which might be applied to research areas that are, respectively, low-, intermediate- and high-cost. Importantly, we excluded from our calculations money allocated non-competitively to large infrastructure (e.g., LHC CERN, High End Computing facilities) and to specialized research institutes (e.g., Euratom, the European Molecular Biology Laboratory), as well as funds for commissioned research. In the case of the U.K., we included the budgets currently being allocated, through peer assessment, to institutions in the form of block grants. Given our estimated baseline rates, we then determined what researchers could pay for in terms of PhD students, Postdocs, travel and equipment.

## Methods

We prepared estimates for the Netherlands, the U.S. and the U.K. Our choice of countries was primarily guided by the availability of relevant data and our familiarity with the funding systems in these countries.

For each country, we first estimated the government funds that are currently being allocated through competitive review processes, excluding, as already mentioned, money currently being allocated non-competitively to large infrastructure and specialized research institutes, as well as funds for commissioned research. Next, we estimated the number of researchers that would qualify for a baseline grant. We only considered researchers who can be assumed to be sufficiently experienced to manage a research budget, namely junior and senior faculty. Simply dividing the first number by the second yields an estimate of the size of the yearly baseline grant, given current funding levels and assuming that all areas of research receive equal financial support. In order to allow for comparisons with current grant sizes, we multiplied this number by five, as research projects commonly have a duration of five years. Additionally, for the Netherlands, we estimated baselines rates according to research area-specific costs. Unfortunately, available data did not allow doing the same for the U.S. and the U.K.

In a second step, we assessed what our estimated baseline grants would allow researchers to pay for. For each country, we estimated the cost of employing PhD students and Postdocs. Subsequently, we estimated the number of PhD students and Postdocs currently employed, on average, per faculty member. Finally, we calculated whether these average employment rates could be achieved given our estimated baseline grants, and how much research money, if any, would be left to be spent on travel and equipment. We did this for individual faculty members as well as for a situation in which groups of five faculty members pool their research money to form small intra- or inter-departmental research teams.

A more detailed description of how the estimates were reached is given below.

### The Netherlands

In 2014, Dutch universities received a total of €707.4 million from competitive government funding provided by research councils: €420 million from the Netherlands Organisation for Scientific Research [18] and €287.4 million from European funding agencies. The latter number was calculated as follows. The Netherlands received €866.3 million in total for the 17 months from January 2014 through to the end of May 2015 [19]. Dividing this total by 17, and multiplying by 12, gives an estimate of what the Netherlands received in 2014, namely €611.5 million. 47% of that went to universities [19], which gives €287.4 million. With an estimated overhead of 7% [20], the total budget to be distributed amounts to €756.9 million.

The Association of Universities in the Netherlands [21] estimates the number of Dutch professors (from assistant to full professor) at 9,702, expressed in full-time equivalents.

Unlike what is common in the U.S. and the U.K., PhD students in the Netherlands are full employees, receive a regular salary for four years and do not need to pay tuition fees. They cost **~**€47.5k per year [22], but when they graduate, the university receives a government bonus of **~**€90k [23]. Since 75% of PhD students successfully complete their degree [24], the average cost of a PhD student in the Netherlands is **~**€30k per year. The cost of a Postdoc amounts to **~**€65k per year [22]. For each faculty member in the Netherlands there are, on average, 0.89 PhD students and 0.41 Postdocs [21].

Subject-specific baseline rates were determined as follows (more details in S1 File). Research in the Netherlands is put into either of the following categories: “Agriculture”, “Physical sciences”, “Technical sciences”, “Health sciences”, “Economics”, “Legal sciences”, “Behavioral and social sciences”, “Linguistic and cultural sciences” and “Various”. We assigned each of those categories to a cost band. Here we followed the cost band classification used by the Higher Education Funding Council for England [25], resulting in the following classification. “Agriculture”, “Physical sciences”, “Technical sciences” and “Health sciences” are high-cost; “Behavioral and social sciences” is intermediate-cost; “Economics”, “Legal sciences”, “Linguistic and cultural sciences” and “Various” are low-cost. For each cost band we then determined the average PhD student and Postdoc employment rates (based on [21]), and calculated, per cost band and for five years, how much money each staff member would need to continue hiring at current employment rates.

Based on this, we calculated how much of the total annual research budget (i.e., €756,9 million) would be left for travel and equipment. That money was then distributed, again, according to cost band, using the cost weights of the Higher Education Funding Council for England (1.6 for high-cost band; 1.3 for intermediate-cost band; 1.0 for low-cost band). Multiplying this number by five, and adding the five-year budget for hiring, gives us, per cost band, the total five-year baseline rate.

### The United States

For the United States, our calculations were for fiscal year 2011 because this is the last year for which dependable staff estimates are available. In 2011, higher education institutes in the U.S. received a total of $40.76 billion from the main U.S, federal funding agencies (including the United States Department of Defense, the Department of Energy, the Department of Health and Human Services, the National Aeronautics and Space Administration, the National Science Foundation, the United States Department of Agriculture), money to be spent on Research & Development (see table 7 in [26]). Research & Development comprises research, equipment, research training, and tuition remission provided to students working on research [27]. This research funding is allocated to academia primarily—90% percent in case of the National Science Foundation [28]—through competitive review processes (see chapter 5, p. 10, in [29]). Since we lack percentages for other federal funding agencies, we apply 90% to the entire budget of $40.76 billion, which yields $36.68 billion. The overhead is estimated at 4% [30], resulting in a total budget of $38.14 billion.

According to the National Center for Education Statistics [31], a total of 344,579 individuals were employed as staff at higher education institutions either primarily for doing research (71,357 individuals) or for doing a combination of instruction, research and/or public service (273,222 individuals). This number thus covers all junior and senior faculty working at U.S. higher education institutions. Importantly, in contrast with the estimate used for the Netherlands, which is calculated in full-time equivalents, the number for the U.S. includes both full-time and part-time staff. We approximated the average fte for part-time staff by the average fte for part-time staff in U.S. medical schools (estimated at 0.65 fte, based on [32]). Since from the total number of 344,579 faculty members 76,153 individuals are employed on a part-time basis [31], the number of U.S. faculty members expressed in full time equivalents can be estimated at 317,925.

Graduate stipends at even the most prestigious colleges in the U.S. are generally, even before taxes, lower than the average gross salary of Dutch PhD students (see the overview in [33]). Since we lack estimates for average stipend size, we can thus use as a conservative estimate the average cost of a Dutch PhD student, i.e. **~**$39k per year. The yearly average cost of a Postdoc in the U.S. is **~**$45k after taxes [34], or **~**$56k before taxes (at a 25% rate, see [35]). In 2011, there were 626,820 students enrolled in master's or doctorate programs [36], some 35% of which were financed through self-support [36]. Assuming that half of these graduate students were enrolled in a doctoral rather than a master’s program, the number of PhD students in need of financial support can be estimated at 203,716. There were 62,639 Postdoctoral researchers in 2011. For each full-time equivalent of the 317,925 faculty members, there are thus on average 0.64 PhD students and 0.2 Postdocs.

### The United Kingdom

In the U.K., there are two types of competitive university research funding. The first type is project funding allocated through peer review competition. Such funding is apportioned by the U.K. Research Councils, National Academies and European funding agencies. In the academic year 2013–2014, U.K. universities received £687 million from European funding agencies [37]. This amounted to 11% of the overall research income of U.K. universities [38]. U.K. universities attracted 24% of their overall research income from the U.K.’s Research Councils [38], which amounts to £1.49 billion. Additionally, £86.5 million came from the U.K.’s National Academies (Royal Society, British Academy, Royal Academy of Engineering) [39]. Given an estimated overhead of 4% [40], the budget to be distributed through peer review competition totals **~**£2.35 billion.

The second type of competitive university research funding comprises institutional funds that are allocated on the basis of periodical research quality assessments. Thus funding is apportioned by the U.K.’s Higher Education Funding Councils. In the academic year 2013–2014, the budget added up to **~**£1.68 billion [39], or **~**£1.74 billion including an overhead of 4%. We decided to include this budget in our calculations because its allocation probably suffers from problems similar to those associated with the allocation of project funds, including the presence of biases in the assessment of institutions, high opportunity costs, and uncertainty about the reliability of research quality indicators [41].

Direct estimates of junior and senior faculty employed at U.K. universities have, to the best of our knowledge, not been published. The Higher Education Statistics Agency reports (see table 2 in [42]) that the pool of academic staff in the U.K. comprises 94,480 individuals doing teaching and research and 45,580 individuals doing research only. However, these numbers also include non-faculty staff, e.g. (senior) research assistants, senior professional staff, technical team leaders, research fellows (e.g., post-doctoral researchers) and so forth (see “Definitions: Staff/Contract level” in [42]). Therefore we used data from England to estimate the fraction of junior and senior faculty among all academic staff. A table produced by the Higher Education Funding Council for England (see tab “Academic contract type” in [43]) provides numbers for academic staff employed at English higher education institutes according to seven contract types and according to the activities “Research only”, “Teaching only”, and “Teaching and research”. Junior and senior faculty correspond to the contract types “Academic leader”, “Professor”, “Senior/Principal Lecturer”, “Lecturer B/Senior Lecturer”. The contract type “Research Assistants” and “Administrative” are excluded for obvious reasons; the contract type “Lecturer A/Lecturer” is left out because it includes, apart from (junior) lecturers, senior professional staff, senior research assistants, and research fellows (such as post-doctoral researchers). For the activity type “Research only”, the fraction of junior and senior faculty over all academic staff is 20%; for the activity type “Teaching and research” the fraction is 88%. If these percentages are applied to the U.K. data, junior and senior faculty at U.K. higher education institutes can be estimated at 92,258 (full- and part-time). Since we lack data on the average fte for part-time staff, we were not able to convert this number into full-time equivalents.

Yearly doctoral stipends on average amount to ~£17k [44], and are tax-free [45]. Based on the salaries provided by the Royal Society’s Newton International Fellowships [46], the average net salary of a Postdoc may be estimated at **~**£24k per year, or at **~**£28k before taxes (at a 20% rate, see [47]). However, we lack figures for the number of postgraduate students enrolled in doctoral programs, as well as figures for the number of Postdocs employed at U.K. universities. We therefore made calculations assuming average employment rates of both the Netherlands and the U.S.

## Results

An overview of our results is presented in Table 1. A more detailed discussion, per country, is provided below.

**Table 1:** Estimated five-year budgets for individual researchers and research teams comprising five faculty members. Budgets for travel and equipment were calculated by subtracting from the total budget the money needed to employ PhD students and Postdocs at current employment levels.

	Individual		Small research team	
	Total five-year budget	Five-year budget for travel and equipment	Total five-year budget	Five-year budget for travel and equipment
**The Netherlands**	**~**$507k	**~**$160k	**~**$2,535k	**~**$800k
**United States**	**~**$599k	**~**$418k	**~**$2,900k	**~**$2,100k
**United Kingdom**	**~**$364k	**~**$143-227k	**~**$1,800k	**~**$717k-1,100k

### The Netherlands

For a typical project duration of 5 years, each Dutch professor (from assistant to full professor) could receive a baseline grant of **~**€390k from what is currently allocated to competitive government funding alone. Given a euro to dollar conversation rate of 1.3 (i.e., the average rate of 2014), this amounts to **~**$507k, or **~**$2.5 million for a research team of five faculty members. Since in the Netherlands the salaries of faculty members are usually covered by their own universities (rather than by grant money), this money could be almost entirely spent on research.

When hiring at current average employment levels of PhD students and Postdocs, an individual faculty member in the Netherlands could thus spend **~**€123k (or **~**$160k) per five years on travel and equipment. A research team (intra- or inter-departmental) comprising five faculty members would have at its disposal a budget of **~**€615k (**~**$800 million) per five years.

Table 2 presents the baseline rates for low-, intermediate- and high-cost disciplines. Despite the fact that researchers working in low-cost disciplines would receive less than they would in the previous scenario, they still would be able to spend in five years ~$118k on travel and equipment—which corresponds to ~$590k, if they were to team up with four other researchers. This amount increases up to **~**$949k for small research teams working in high-cost disciplines.

**Table 2:** Estimated five-year budgets, per cost band, for individual researchers and research teams comprising five faculty members (the Netherlands). Budgets for travel and equipment were calculated assuming current PhD student and Postdoc employment levels.

	Individual		Small research team	
	Total five-year budget	Five-year budget for travel and equipment	Total five-year budget	Five-year budget for travel and equipment
**Low-cost**	**~**$290k	**~**$118k	**~**$1,450k	**~**$590k
**Intermediate-cost**	**~**$455k	**~**$154k	**~**$2,275k	**~**$770k
**High-cost**	**~**$715k	**~**$189k	**~**$3,575k	**~**$945k

### The United States

In the U.S., each faculty member could be given a baseline grant of **~**$553k over a period of five years. The size of the grant increases to **~**$599k when the total available budget is distributed according to full-time equivalents. As in the Netherlands, the salaries of U.S. faculty members are usually paid by their universities.

A research team of five faculty members could, in addition to being able to maintain PhD student and Postdoc numbers at current employment rates, spend **~**$2.1 million per five years on travel and equipment, which amounts to **~**$418k per individual faculty member (both numbers calculated relying on staff numbers expressed in full-time equivalents).

### The United Kingdom

If the budgets from the two types of competitive funding in the U.K. are combined, all junior and senior faculty could be given a baseline grant of **~**£221k for five years, which, given a conversion rate of 1.65 (i.e., the average rate for the academic year 2013/14), amounts to **~**$364k. This baseline breaks down into **~**£127k (**~**$209k) coming from what is currently allocated based on grant peer review (first type), and **~**£94k (**~**$155k) coming from what is currently allocated based on periodical research quality assessments by U.K.’s Higher Education Funding Councils (second type).

If we set PhD/faculty and Postdoc/faculty rates at the level of the Netherlands, a five faculty research team would have a working budget of **~**£435k (or ~$717k) per five years; individual staff members could spend **~**£87k (or ~$143k). Assuming U.S. rates, the team’s budget would amount to **~**£690k (or **~**$1.1 million) and an individual faculty member’s budget to **~**£138k (**~**$227k).

One complication, however, is that in the U.K., unlike in the Netherlands and the U.S., universities might choose how to spend money received from the aforementioned second type of competitive funding (i.e., institutional funds allocated based on periodical research quality assessments). Plausibly, this implies that institutions spend part of the budget they obtain from the Higher Education Funding Councils on faculty members’ salaries. Consequently, a fraction of our estimate for this type of funding, namely a fraction of **~**£94k (**~**$155k) per faculty member, might not serve as a supplement to faculty members’ salaries.

## Discussion

Obviously, the level of funding in a baseline grant system would be lower than the level of funding in, for example, the most generous personal grant schemes awarded in the Netherlands (€1.5 million for NWO’s Vici Grants), Europe (€2.5 million for ERC’s Advanced Grants) and the U.S. ($1.7 million for NIH’s R01 Grants). However, such personal grants are awarded to only 10–15% of applicants. Thus whether the same percentage of individuals continue to receive similar funds is not a benchmark for the overall research performance of countries; we need, instead, to consider the overall impact of proposed redistributions of funds. A more meaningful assessment is therefore to consider, as we have done above, *at the country level* whether our estimated baseline grants would allow individual researchers and small research teams to employ as many PhD students and Postdocs as they currently do, and whether the money left for travel and equipment would remain substantial. In the Netherlands and the U.S., if faculty members’ salaries continue to be paid by their universities, the answer to the first question appears on average to be “yes”. Further, our calculations for the Netherlands suggest that by applying subject-specific baseline rates, researchers working in high-cost disciplines might be able to invest quite a bit in costly equipment. The baseline rates in the U.K. would be substantially lower than those in the Netherlands and the U.S. (but recall that they were calculated in terms of absolute numbers of faculty members rather than in terms of full-time equivalents). Additionally, part of that lower baseline income would, plausibly, need to be allocated to faculty members’ salaries. So for the U.K. the worry concerning dilution of resources seems, at least in light of the currently available evidence, justified (but see below).

## Conclusion

While our estimated budgets for travel and equipment seem substantial (at least so for the Netherlands and the U.S.), they might in the end turn out to be insufficient. For the Netherlands we have used discipline-specific cost rates from the U.K. These rates might underestimate the costs associated with resource-intensive research in the Netherlands and, perhaps, if appropriate cost rates were applied to the U.S. and the U.K., resources there would be diluted too much. On the other hand, we may have, to some extent, underestimated potential budgets under egalitarian sharing of the kind we have been considering. One should, for example, add to our estimates resources currently being spent on peer assessment. By way of illustration, it has been estimated that, for the Australian National Health and Medical Research Council alone, researchers in 2012 lost about 400 years of research time in writing unsuccessful proposals [2]. Regarding the resources spent on reviewing, in 2014 the US National Science Foundation received a total of 48,051 proposals, 96% of which were evaluated by reviewers as well as by the Foundation’s staff [48]. In the period 2005/06, reviewers for the UK Research Councils can be estimated to have spent 192 years on assessing applications [40]. Also, the money currently being spent on consultants and internal project administration might be added to institutions’ research budgets. For a more complete analysis than we have provided, further study will thus have to estimate, per discipline, the total research budget available to researchers (i.e., summing their baseline grant, non-competitive money and resources freed up by not having to write and review grant proposals) and, subsequently, set such budgets against effective research costs. Even the moderate baseline rates of the U.K. might turn out to be sufficiently large in light of such an analysis, especially if they were differentiated according to subject-specific costs.

Note that even if dilution of resources in the end were to appear problematic, our results suggest that it is feasible to distribute funds more widely than is the case in current competitive funding schemes, many of which allocate large sums of money only to a select “excellent” few (with success rates of as low as 10–15%). With a success rate of 60%, for example, a research team of five members could, in addition to employing PhD students and Postdocs at current employment levels, spend per five years on travel and equipment a budget of **~**$2.9 million in the Netherlands, of ~$4.1 million in the U.S., and of ~$1.9–2.3 million in the U.K. If one were to differentiate according to subject-specific costs, small research teams working in high-cost disciplines (the Netherlands) would have at their disposal **~**$2,525k for travel and equipment (see Table 3).

**Table 3:** Estimated five-year budgets, per cost band, for individual researchers and research teams comprising five faculty members, when 60% of researchers would receive a baseline grant (the Netherlands). Budgets for travel and equipment were calculated assuming current PhD student and Postdoc employment levels.

	Individual		Small research team	
	Total five-year budget	Five-year budget for travel and equipment	Total five-year budget	Five-year budget for travel and equipment
**Low-cost**	**~**$487k	**~**$316k	**~**$2,435k	**~**$1,580k
**Intermediate-cost**	**~**$711k	**~**$410k	**~**$3,555k	**~**$2,050k
**High-cost**	**~**$1,031k	**~**$505k	**~**$5,155k	**~**$2,525k

In order to keep opportunity costs to a bare minimum, the selection of this 60% could be based not on evaluations of proposals, but on (largely automated) evaluations of an applicant’s research track record (as suggested by [11–12]). Although past achievement is not known to be a reliable proxy of future success, assessing scientists on what they do anyway looks like the solution that would cause the least damage.

Obviously, there are other objections one might raise against egalitarian sharing, and these would need to be addressed too in order to fully assess its feasibility. One would like to address the worry that a baseline grant system has insufficient checks and balances, and thus that such a system suffers from problems of accountability towards taxpayers. There are various reasons not to think of this as a serious worry—research money could be allocated solely to tenure-track or tenured researchers whose salaries would be paid from universities’ own resources (rather than by funding agencies), scholarly work would be certified in the usual ways, various external checks of researchers’ and departments’ financial responsibility are already in place—but we do agree it deserves further attention. Further, there is the question of the extent to which grant-giving bodies should relinquish some of their control over the course of science. Here, one should keep in mind that while control over the societal relevance of science may diminish if no compensating measures are put in place, less control over which research is carried out may contribute to an increase in the diversity of research programs and thus to progress. Finally, one might worry that talented researchers would drop out of science in the absence of strong incentives to excel—perhaps as many researchers as under strong competition. By the same token, however, one might worry that rewarding only a select few means that unutilized talent is dropping out of science. These issues do need to be sorted out. But, as we have seen, current schemes face serious problems, and there has been limited investigation of how they compare to egalitarian approaches. Given that egalitarian sharing has potential advantages, that the worry that egalitarian sharing leads to unacceptable dilution of resources appears to be unjustified and that there appears to be room for far more egalitarian sharing than is currently the norm, egalitarian approaches deserve to be taken seriously.

## Supporting information

S1 File Calculation of subject-specific baseline rates for the Netherlands. (RTF)
