# Supplementary material for: How much would each researcher receive if competitive government research funding were distributed equally among researchers?
Source: PLoS One. 2017 Sep 8;12(9):e0183967. doi: 10.1371/journal.pone.0183967 (PMC5590858; doi:10.1371/journal.pone.0183967)
Supplement: S1 File — (RTF) [file pone.0183967.s001.rtf]

S1 File: Calculation of subject-specific baseline rates for the Netherlands
In a first step, we assigned subjects to a cost band. Subsequently we calculated subject-specific baseline rates comprising two parts, namely a budget for hiring at current subject-specific PhD student and Postdoc employment rates, and a subject-specific budget for travel and equipment. 

Assigning subjects to a cost band
Research in the Netherlands falls under one of the following categories: “Agriculture”, “Physical sciences”, “Technical sciences”, “Health sciences”, “Economics”, “Legal sciences”, “Behavioral and social sciences”, “Linguistic and cultural sciences” and “Various”. We relied on a classification of the Higher Education Funding Council for England (HEFCE) [24] to assign these categories to three cost bands, respectively, low-, intermediate- and high-cost. S1 Table represents the way in which we matched the HEFCE categories to those of the Netherlands. We assigned to each Netherlands' subject area the cost band of the majority of the corresponding HEFCE subject areas.

S1 Table: HEFCE subject areas, HEFCE cost bands, subject areas in the Netherlands, and assigned cost bands for the Netherlands.
HEFCE Subject Area	HEFCE
Cost
Band	Subject Area
Netherlands	Cost
Band Netherlands (assigned)	
Cardiovascular Medicine	H 	Health Sciences	H	
Cancer Studies	H 	Health Sciences	H	
Infection and Immunology	H 	Health Sciences	H	
Other Hospital Based Clinical Subjects	H 	Health Sciences	H	
Other Laboratory Based Clinical Subjects	H 	Health Sciences	H	
Epidemiology and Public Health	H 	Health Sciences	H	
Health Services Research	H 	Health Sciences	H	
Primary Care and Other Community Based Clinical Subjects	H 	Health Sciences	H	
Psychiatry, Neuroscience and Clinical Psychology	H 	Health Sciences	H	
Dentistry	H 	Health Sciences	H	
Nursing and Midwifery	I 	Health Sciences	H	
Allied Health Professions and Studies	H 	Health Sciences	H	
Pharmacy	H 	Health Sciences	H	
Biological Sciences	H 	Physical Sciences	H	
Pre-clinical and Human Biological Sciences	H 	Health Sciences	H	
Agriculture, Veterinary and Food Science	H 	Agriculture	H	
Earth Systems and Environmental Sciences	H 	Physical Sciences	H	
Chemistry	H 	Physical Sciences	H	
Physics	H 	Physical Sciences	H	
Pure Mathematics	I 	Physical Sciences	H	
Applied Mathematics	H 	Physical Sciences	H	
Statistics and Operational Research	H 	Physical Sciences	H	
Computer Science and Informatics	H 	Technical Sciences	H	
Electrical and Electronic Engineering	H 	Technical Sciences	H	
General Engineering and Mineral & Mining Engineering	H 	Technical Sciences	H	
Chemical Engineering	H 	Technical Sciences	H	
Civil Engineering	H 	Technical Sciences	H	
Mechanical, Aeronautical and Manufacturing Engineering	H 	Technical Sciences	H	
Metallurgy and Materials	H 	Technical Sciences	H	
Architecture and the Built Environment	I 	Technical Sciences	H	
Town and Country Planning	I 	Technical Sciences	H	
Geography and Environmental Studies	I 	Physical Sciences	H	
Archaeology	I 	Behavioral and social sciences	I	
Economics and Econometrics	L 	Economics	L	
Accounting and Finance	L 	Economics	L	
Business and Management Studies	L 	Economics	L	
Library and Information Management	L 	Various	L	
Law	L 	Legal Sciences	L	
Politics and International Studies	L 	Behavioral and social sciences	I	
Social Work and Social Policy & Administration	L 	Behavioral and social sciences	I	
Sociology	L 	Behavioral and social sciences	I	
Anthropology	L 	Behavioral and social sciences	I	
Development Studies	L 	Behavioral and social sciences	I	
Psychology	I 	Behavioral and social sciences	I	
Education	L 	Behavioral and social sciences	I	
Sports-Related Studies	I 	Behavioral and social sciences	I	
American Studies and Anglophone Area Studies	L 	Linguistic and Cultural Studies	L	
Middle Eastern and African Studies	L 	Linguistic and Cultural Studies	L	
Asian Studies	L 	Linguistic and Cultural Studies	L	
European Studies	L 	Linguistic and Cultural Studies	L	
Russian, Slavonic and East European Languages	L 	Linguistic and Cultural Studies	L	
French	L 	Linguistic and Cultural Studies	L	
German, Dutch and Scandinavian Languages	L 	Linguistic and Cultural Studies	L	
Italian	L 	Linguistic and Cultural Studies	L	
Iberian and Latin American Languages	L 	Linguistic and Cultural Studies	L	
Celtic Studies	L 	Linguistic and Cultural Studies	L	
English Language and Literature	L 	Linguistic and Cultural Studies	L	
Linguistics	L 	Linguistic and Cultural Studies	L	
Classics, Ancient History, Byzantine and Modern Greek Studies	L 	Linguistic and Cultural Studies	L	
Philosophy	L 	Linguistic and Cultural Studies	L	
Theology, Divinity and Religious Studies	L 	Linguistic and Cultural Studies	L	
History	L 	Linguistic and Cultural Studies	L	
Art and Design	I 	Linguistic and Cultural Studies	L	
History of Art, Architecture and Design	L 	Linguistic and Cultural Studies	L	
Drama, Dance and Performing Arts	I 	Linguistic and Cultural Studies	L	
Communication, Cultural and Media Studies	L 	Linguistic and Cultural Studies	L	
Music	I 	Linguistic and Cultural Studies	L	


Calculation of budgets for hiring at current employment rates
For each of the cost bands, and based on [20], we calculated the average current PhD student and Postdoc employment rates. Given the average annual cost of PhD students (i.e., ~€30k) and of Postdocs (i.e., ~€65k), we could then, per cost band, estimate the annual budget researchers need in order to hire at current employment rates. See S2 Table.

S2 Table: Employment rates and annual individual budget for hiring per cost band
	Average PhD employment rate	Average Postdoc employment rate	Total annual individual budget for hiring	
Low-cost	0.49	0.18	€26,400	
Intermediate-cost	0.72	0.38	€46,300	
High-cost	1.33	0.63	€80,850	

Calculation of budgets for travel and equipment
Multiplying the annual individual budget for hiring by the number of staff members per cost band (see S3 Table) gives us the total Dutch budget for hiring PhD students and Postdocs. Subtracting this amount from the total Dutch research budget estimated in the main text (i.e., €885.3 million) yields the total budget that can be spent on travel and equipment (i.e., ~€388.2 million). This budget was then distributed according to the number of staff members working in each cost band, and applying a subject specific cost-weight. The result is the annual subject-specific individual budget for travel and equipment.

S3 Table: Number of staff members, cost-weight factor and total individual budget for travel and equipment per cost band
	Number of staff members	Cost-weight	Total annual individual budget for travel and equipment	
Low-cost	3,578	1.0	€28,300	
Intermediate-cost	1,922	1.3	€36,791	
High-cost	4,201	1.6	€45,281	

Total five-year subject-specific baseline rates
Summing the total individual annual budgets for hiring and travel and equipment, multiplying them by five, and applying a euro to dollar conversion rate of 1.3, yields the numbers in the second column of Table 2 in the main text. 
